# Correction: Health disparities in cervical cancer: Estimating geographic variations of disease burden and association with key socioeconomic and demographic factors in the US

**DOI:** 10.1371/journal.pone.0324971

**Published:** 2025-05-20

**Authors:** Tara Castellano, Andrew K. ElHabr, Christina Washington, Jie Ting, Yitong J. Zhang, Fernanda Musa, Ezgi Berksoy, Kathleen Moore, Leslie Randall, Jagpreet Chhatwal, Turgay Ayer, Charles A. Leath

The Funding statement for this article is incorrect. The correct Funding statement is as follows: This study was funded by Seagen Inc., which was acquired by Pfizer Inc., in December 2023, and Genmab A/S. The funders had a role in the study design and decision to publish this article.

In the Competing Interests section, there are typographical errors in the first sentence. The correct sentence is: Tara Castellano has received consulting fees from Glaxo-Smith-Kline; Andrew K. ElHabr and Ezgi Berksoy are paid employees of Value Analytics Labs, a healthcare consultancy company; Jie Ting and Yitong J. Zhang are employees and stock holders of Pfizer Inc.; Kathleen Moore has participated in data monitoring or advisory boards for Astra Zeneca, Aravive, Alkemeres, Aadi, Blueprint pharma, Clovis, Caris, Duality, Elevar, Eisai, EMD Serono, GSK/Tesaro, Genentech/Roche, Hengrui, Immunogen, Janssen, Lilly, Mersana, Merck, Myriad, Mereo, Novartis, OncXerna, Onconova, SQZ, Tarveda, VBL Therapeutics, Verastem and Zentalis, received support for attending meetings from Astra Zeneca, GSK/Tesaro, and BioNTech, and holds a leadership role with GOG partners; Leslie Randall reports personal fees from Seagen Inc. for an educational webinar at drug launch and speaker’s bureau and personal fees from Merck for an unbranded educational video for cervical cancer.
