# Supplementary material for: Health disparities in cervical cancer: Estimating geographic variations of disease burden and association with key socioeconomic and demographic factors in the US
Source: PLoS One. 2024 Jul 18;19(7):e0307282. doi: 10.1371/journal.pone.0307282 (PMC11257296; doi:10.1371/journal.pone.0307282)
Supplement: S1 Table — (DOCX) [file pone.0307282.s001.docx]

| **Region** | **States** |
| --- | --- |
| Midwest | Michigan, Iowa, Illinois, Indiana, Kansas, Michigan, Minnesota, Missouri, North Dakota, Nebraska, Ohio, South Dakota, Wisconsin |
| Northeast | Connecticut, Massachusetts, Maine, New Hampshire, New Jersey, New York, Pennsylvania, Rhode Island, Vermont |
| South | Alabama, Arkansas, District of Columbia, Delaware, Florida, Georgia, Kentucky, Louisiana, Maryland, Mississippi, North Carolina, Oklahoma, South Carolina, Tennessee, Texas, Virginia, West Virginia |
| West | Alaska, Arizona, California, Colorado, Hawaii, Idaho, Montana, New Mexico, Nevada, Oregon, Utah, Washington, Wyoming |
