# Supplementary material for: Health disparities in cervical cancer: Estimating geographic variations of disease burden and association with key socioeconomic and demographic factors in the US
Source: PLoS One. 2024 Jul 18;19(7):e0307282. doi: 10.1371/journal.pone.0307282 (PMC11257296; doi:10.1371/journal.pone.0307282)
Supplement: S2 Table — (DOCX) [file pone.0307282.s002.docx]

| **Year** | **Screening-Eligible Enrollees^a^** | **% Screened^a^** | **Prevalent CC Count** | **CC Burden** | **r/mCC Burden** |
| --- | --- | --- | --- | --- | --- |
| 2017 | - | - | 27,187 | 84 | 6% |
| 2018 | - | - | 30,010 | 99 | 6% |
| 2019 | 21,924,103 | 56% | 31,868 | 109 | 6% |
| 2020 | 24,585,338 | 54% | 32,354 | 95 | 6% |
| 2021 | 24,927,973 | 55% | 33,705 | 97 | 5% |
| 2022 | 25,390,023 | 53% | 31,805 | 90 | 6% |

CC, cervical cancer; r/mCC, recurrent or metastatic cervical cancer.

^a^ Numbers are not recorded for 2017-2018 to allow for full 5-year histories of eligible enrollees to be evaluated.
