# Supplementary material for: Health disparities in cervical cancer: Estimating geographic variations of disease burden and association with key socioeconomic and demographic factors in the US
Source: PLoS One. 2024 Jul 18;19(7):e0307282. doi: 10.1371/journal.pone.0307282 (PMC11257296; doi:10.1371/journal.pone.0307282)
Supplement: S3 Table — (DOCX) [file pone.0307282.s003.docx]

| **Univariate Regression Description** | **Coefficient (95% Confidence Interval)** | **Standard Error** | **p-value** | **R^2^** |
| --- | --- | --- | --- | --- |
| Screening rate vs. CC burden: South (Figure 2A) | -1.63 (-2.38, -0.88) | 0.38 | <0.001 | 0.057 |
| Screening rate vs. r/mCC burden: South (Figure 2B) | -0.25 (-0.45, -0.05) | 0.10 | 0.013 | 0.025 |
| Screening rate vs. r/mCC burden: Midwest (Figure 2B) | -0.30 (-0.53, -0.08) | 0.11 | 0.009 | 0.045 |
| Screening rate vs. r/mCC burden: West (Figure 2B) | 0.25 (0.01, 0.49) | 0.12 | 0.038 | 0.040 |
| Screening rate vs. CC burden: West (Figure 2A) | 1.04 (-0.02, 2.10) | 0.54 | 0.055 | 0.023 |
| Percentage of low-income households vs. CC burden: All (Figure 3A) | 1.74 (1.50, 1.98) | 0.12 | <0.001 | 0.190 |
| Percentage of low-income households vs. r/mCC burden: South (Figure 3B) | 0.21 (0.08, 0.33) | 0.06 | 0.001 | 0.043 |
| Proportion of Hispanic population vs. CC burden: All (Figure 4) | 0.27 (0.13, 0.41) | 0.07 | <0.001 | 0.016 |
| Proportion of Asian population vs. CC burden: All (Figure 4) | -0.98 (-1.38, -0.58) | 0.20 | <0.001 | 0.026 |
| Proportion of Hispanic population vs. CC burden: West (Figure 4) | 0.75 (0.44, 1.07) | 0.16 | <0.001 | 0.125 |
| Proportion of Asian population vs. CC burden: South (Figure 4) | -4.40 (-5.67, -3.13) | 0.64 | <0.001 | 0.132 |
| Proportion of Asian population vs. r/mCC burden: Midwest (Figure 4) | -0.61 (-1.21, -0.01) | 0.30 | 0.047 | 0.027 |
| Proportion of Black population vs. CC burden: Midwest (Figure 4) | 0.82 (0.37, 1.26) | 0.22 | <0.001 | 0.056 |
| Proportion of Black population vs. CC burden: Northeast (Figure 4) | 0.90 (0.38, 1.43) | 0.27 | <0.001 | 0.065 |
| Proportion of Black population vs. CC burden: South (Figure 4) | -0.53 (-0.77, -0.30) | 0.12 | <0.001 | 0.063 |
| Presence of a brachytherapy center vs. r/mCC burden: All (Figure 5) | -2.78 (-3.89, -1.67) | 0.56 | <0.001 | 0.036 |
| Presence of a brachytherapy center vs. r/mCC burden: Midwest | -4.40 (-7.1, -1.7) | 1.36 | 0.001 | 0.066 |
| Presence of a brachytherapy center vs. r/mCC burden: South | -3.02 (-4.80, -1.24) | 0.90 | <0.001 | 0.044 |
