# Supplementary figures and images for: Health disparities in cervical cancer: Estimating geographic variations of disease burden and association with key socioeconomic and demographic factors in the US
Source: PLoS One. 2024 Jul 18;19(7):e0307282. doi: 10.1371/journal.pone.0307282 (PMC11257296; doi:10.1371/journal.pone.0307282)

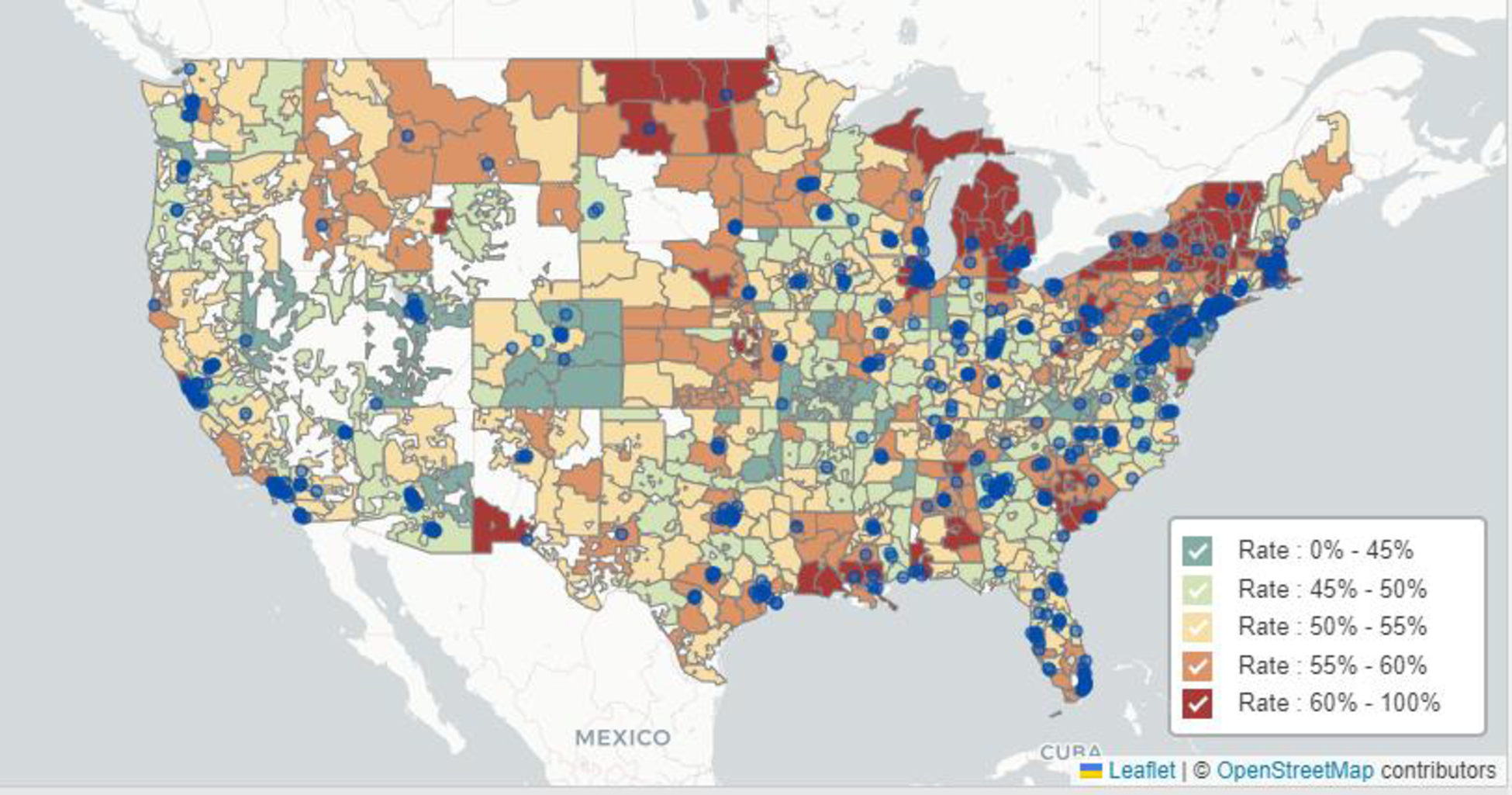

Supplement: S1 Fig — A patient was considered screened for cervical cancer during a year of interest if she was either between the ages of 21 and 64 and had cervical cytology performed within the previous three years, or between the ages of 30 and 64 and had cervical hrHPV testing performed with or without cytology within the previous five years. Contains information from OpenStreetMap and OpenStreetMap Foundation, which is licensed under the Open Data Commons Open Database License (https://www.openstreetmap.org/copyright). (TIF) [file pone.0307282.s004.tif]
