# Supplementary material for: Health disparities in cervical cancer: Estimating geographic variations of disease burden and association with key socioeconomic and demographic factors in the US
Source: PLoS One. 2024 Jul 18;19(7):e0307282. doi: 10.1371/journal.pone.0307282 (PMC11257296; doi:10.1371/journal.pone.0307282)
Supplement: Attachment — Submitted filename: reviewer-responses.docx [file pone.0307282.s005.docx]

**Response to reviewers**

When submitting your revision, we need you to address these additional requirements.

1. Please ensure that your manuscript meets PLOS ONE's style requirements, including those for file naming. The PLOS ONE style templates can be found at 
[https://journals.plos.org/plosone/s/file?id=wjVg/PLOSOne_formatting_sample_main_body.pdf](https://urldefense.com/v3/__https:/journals.plos.org/plosone/s/file?id=wjVg*PLOSOne_formatting_sample_main_body.pdf__;Lw!!NoSwA-eRAg!BtSAgaunO-NI9ok45AiYVMTB9SM7Td4gjwiYC6wu7oGyluhSgTvolsnci87HjQeD8tfyYqHuLN7tCiWsso8fT8eJdAAW-GYL$) and 
[https://journals.plos.org/plosone/s/file?id=ba62/PLOSOne_formatting_sample_title_authors_affiliations.pdf](https://urldefense.com/v3/__https:/journals.plos.org/plosone/s/file?id=ba62*PLOSOne_formatting_sample_title_authors_affiliations.pdf__;Lw!!NoSwA-eRAg!BtSAgaunO-NI9ok45AiYVMTB9SM7Td4gjwiYC6wu7oGyluhSgTvolsnci87HjQeD8tfyYqHuLN7tCiWsso8fT8eJdPlLb49H$)

Thank you for the reminder. We have done our best to update the affiliations formatting and general text formatting to follow these directions now. We have also converted all figure files into .tif files to comply with journal guidelines.

2. Thank you for stating the following in the Competing Interests section: 
"Tara Castellano has received consulting fees from Glaxo-Smith-Klein; Andrew K. ElHabr and Ezgi Berksoy are paid employees of Value Analytics Labs, a healthcare consultancy company; Jie Ting and Yitong J. Zhang are employees and stack holders of Pfizer Inc.; Kathleen Moore has participated in data monitoring or advisory boards for Astra Zeneca, Aravive, Alkemeres, Aadi, Blueprint pharma, Clovis, Caris, Duality, Elevar, Eisai, EMD Serono, GSK/Tesaro, Genentech/Roche, Hengrui, Immunogen, Janssen, Lilly, Mersana, Merck, Myriad, Mereo, Novartis, OncXerna, Onconova, SQZ, Tarveda, VBL Therapeutics, Verastem and Zentalis, received support for attending meetings from Astra Zeneca, GSK/Tesaro, and BioNTech, and holds a leadership role with GOG partners; Leslie Randall reports personal fees from Seagen Inc. for an educational webinar at drug launch and speaker's bureau and personal fees from Merck for an unbranded educational video for cervical cancer. Her institute receives research funding for clinical research from Seagen and Merck. She reports personal fees from BluPrint Oncology, PER, CurioScience, Projects in Knowledge, AstraZeneca, Tesaro, Merck, Mersana, Agenus, Rubius Therapeutics, Myriad Genetics, EMD Serono, Genentech/Roche, Seattle Genetics, Novartis, and Eisai, all outside the submitted work; Jagpreet Chhatwal and Turgay Ayer are co-owners of Value Analytics Labs; Charles A. Leath III has received consulting fees from Seagen Inc. for service on Scientific Advisory boards, cervical cancer research funding from Agenus, Rubius Therapeutics, and Seagen Inc., and funding from the NCI UG1 CA23330 and P50 CA098252; Fernanda Musa and Christina Washington have no competing interests to disclose."

Please confirm that this does not alter your adherence to all PLOS ONE policies on sharing data and materials, by including the following statement: ""This does not alter our adherence to  PLOS ONE policies on sharing data and materials.” (as detailed online in our guide for authors [http://journals.plos.org/plosone/s/competing-interests).](https://urldefense.com/v3/__http:/journals.plos.org/plosone/s/competing-interests).__;!!NoSwA-eRAg!BtSAgaunO-NI9ok45AiYVMTB9SM7Td4gjwiYC6wu7oGyluhSgTvolsnci87HjQeD8tfyYqHuLN7tCiWsso8fT8eJdBWxqRs7$)  If there are restrictions on sharing of data and/or materials, please state these. Please note that we cannot proceed with consideration of your article until this information has been declared.

Please include your updated Competing Interests statement in your cover letter; we will change the online submission form on your behalf.

Thank you. We have added our original Competing Interests statement and the following statement to our cover letter now:

*“These competing interests do not alter our adherence to PLOS ONE policies on sharing data and materials.”*

3. When completing the data availability statement of the submission form, you indicated that you will make your data available on acceptance. We strongly recommend all authors decide on a data sharing plan before acceptance, as the process can be lengthy and hold up publication timelines. Please note that, though access restrictions are acceptable now, your entire data will need to be made freely accessible if your manuscript is accepted for publication. This policy applies to all data except where public deposition would breach compliance with the protocol approved by your research ethics board. If you are unable to adhere to our open data policy, please kindly revise your statement to explain your reasoning and we will seek the editor's input on an exemption. Please be assured that, once you have provided your new statement, the assessment of your exemption will not hold up the peer review process.

The data are now uploaded to the public repository: <https://github.com/valueanalyticslabs/geoanalyzer-public>. The data availability statement has been revised accordingly:

*“The data used to generate the findings in this study can be found at* [*https://github.com/valueanalyticslabs/geoanalyzer-public*](https://github.com/valueanalyticslabs/geoanalyzer-public)*.”*

4. We note that Figure 1 and S1 in your submission contain map/satellite images which may be copyrighted. All PLOS content is published under the Creative Commons Attribution License (CC BY 4.0), which means that the manuscript, images, and Supporting Information files will be freely available online, and any third party is permitted to access, download, copy, distribute, and use these materials in any way, even commercially, with proper attribution. For these reasons, we cannot publish previously copyrighted maps or satellite images created using proprietary data, such as Google software (Google Maps, Street View, and Earth). For more information, see our copyright guidelines: [http://journals.plos.org/plosone/s/licenses-and-copyright](https://urldefense.com/v3/__http:/journals.plos.org/plosone/s/licenses-and-copyright__;!!NoSwA-eRAg!BtSAgaunO-NI9ok45AiYVMTB9SM7Td4gjwiYC6wu7oGyluhSgTvolsnci87HjQeD8tfyYqHuLN7tCiWsso8fT8eJdFDyrh__$).
 
We require you to either (1) present written permission from the copyright holder to publish these figures specifically under the CC BY 4.0 license, or (2) remove the figures from your submission:
 
a. You may seek permission from the original copyright holder of Figure 1 and S1 to publish the content specifically under the CC BY 4.0 license.  
 
We recommend that you contact the original copyright holder with the Content Permission Form ([http://journals.plos.org/plosone/s/file?id=7c09/content-permission-form.pdf](https://urldefense.com/v3/__http:/journals.plos.org/plosone/s/file?id=7c09*content-permission-form.pdf__;Lw!!NoSwA-eRAg!BtSAgaunO-NI9ok45AiYVMTB9SM7Td4gjwiYC6wu7oGyluhSgTvolsnci87HjQeD8tfyYqHuLN7tCiWsso8fT8eJdBkAAtq-$)) and the following text:
“I request permission for the open-access journal PLOS ONE to publish XXX under the Creative Commons Attribution License (CCAL) CC BY 4.0 ([http://creativecommons.org/licenses/by/4.0/](https://urldefense.com/v3/__http:/creativecommons.org/licenses/by/4.0/__;!!NoSwA-eRAg!BtSAgaunO-NI9ok45AiYVMTB9SM7Td4gjwiYC6wu7oGyluhSgTvolsnci87HjQeD8tfyYqHuLN7tCiWsso8fT8eJdMdWz7bD$)). Please be aware that this license allows unrestricted use and distribution, even commercially, by third parties. Please reply and provide explicit written permission to publish XXX under a CC BY license and complete the attached form.”
 
Please upload the completed Content Permission Form or other proof of granted permissions as an ""Other"" file with your submission.
 
In the figure caption of the copyrighted figure, please include the following text: “Reprinted from [ref] under a CC BY license, with permission from [name of publisher], original copyright [original copyright year].”
 
b. If you are unable to obtain permission from the original copyright holder to publish these figures under the CC BY 4.0 license or if the copyright holder’s requirements are incompatible with the CC BY 4.0 license, please either i) remove the figure or ii) supply a replacement figure that complies with the CC BY 4.0 license. Please check copyright information on all replacement figures and update the figure caption with source information. If applicable, please specify in the figure caption text when a figure is similar but not identical to the original image and is therefore for illustrative purposes only.
The following resources for replacing copyrighted map figures may be helpful:
 
USGS National Map Viewer (public domain): [http://viewer.nationalmap.gov/viewer/](https://urldefense.com/v3/__http:/viewer.nationalmap.gov/viewer/__;!!NoSwA-eRAg!BtSAgaunO-NI9ok45AiYVMTB9SM7Td4gjwiYC6wu7oGyluhSgTvolsnci87HjQeD8tfyYqHuLN7tCiWsso8fT8eJdMzq5eOk$)
The Gateway to Astronaut Photography of Earth (public domain): [http://eol.jsc.nasa.gov/sseop/clickmap/](https://urldefense.com/v3/__http:/eol.jsc.nasa.gov/sseop/clickmap/__;!!NoSwA-eRAg!BtSAgaunO-NI9ok45AiYVMTB9SM7Td4gjwiYC6wu7oGyluhSgTvolsnci87HjQeD8tfyYqHuLN7tCiWsso8fT8eJdHkZ_l2Q$)
Maps at the CIA (public domain): [https://www.cia.gov/library/publications/the-world-factbook/index.html](https://urldefense.com/v3/__https:/www.cia.gov/library/publications/the-world-factbook/index.html__;!!NoSwA-eRAg!BtSAgaunO-NI9ok45AiYVMTB9SM7Td4gjwiYC6wu7oGyluhSgTvolsnci87HjQeD8tfyYqHuLN7tCiWsso8fT8eJdNBFWglu$) and [https://www.cia.gov/library/publications/cia-maps-publications/index.html](https://urldefense.com/v3/__https:/www.cia.gov/library/publications/cia-maps-publications/index.html__;!!NoSwA-eRAg!BtSAgaunO-NI9ok45AiYVMTB9SM7Td4gjwiYC6wu7oGyluhSgTvolsnci87HjQeD8tfyYqHuLN7tCiWsso8fT8eJdFjeemQW$)
NASA Earth Observatory (public domain): [http://earthobservatory.nasa.gov/](https://urldefense.com/v3/__http:/earthobservatory.nasa.gov/__;!!NoSwA-eRAg!BtSAgaunO-NI9ok45AiYVMTB9SM7Td4gjwiYC6wu7oGyluhSgTvolsnci87HjQeD8tfyYqHuLN7tCiWsso8fT8eJdGNmpUzD$)
Landsat: [http://landsat.visibleearth.nasa.gov/](https://urldefense.com/v3/__http:/landsat.visibleearth.nasa.gov/__;!!NoSwA-eRAg!BtSAgaunO-NI9ok45AiYVMTB9SM7Td4gjwiYC6wu7oGyluhSgTvolsnci87HjQeD8tfyYqHuLN7tCiWsso8fT8eJdEstQD39$)
USGS EROS (Earth Resources Observatory and Science (EROS) Center) (public domain): [http://eros.usgs.gov/#](https://urldefense.com/v3/__http:/eros.usgs.gov/*__;Iw!!NoSwA-eRAg!BtSAgaunO-NI9ok45AiYVMTB9SM7Td4gjwiYC6wu7oGyluhSgTvolsnci87HjQeD8tfyYqHuLN7tCiWsso8fT8eJdDBFdC1C$)
Natural Earth (public domain): [http://www.naturalearthdata.com/](https://urldefense.com/v3/__http:/www.naturalearthdata.com/__;!!NoSwA-eRAg!BtSAgaunO-NI9ok45AiYVMTB9SM7Td4gjwiYC6wu7oGyluhSgTvolsnci87HjQeD8tfyYqHuLN7tCiWsso8fT8eJdJUjYun9$)

Thank you for all of the information and assistance. We used OpenStreetMap as part of the tool to generate Figs 1 and S1. According to them, we are free to copy, distribute, transmit and adapt their data as long as we credit OpenStreetMap and its contributors (<https://www.openstreetmap.org/copyright>). We have now included their copyright notice as part of Figs 1 and S1 and have added that OpenStreetMap is licensed under the Open Data Commons Open Database License (<https://www.openstreetmap.org/copyright>) in the figure captions with the following statement:

*“**Contains information from OpenStreetMap and OpenStreetMap Foundation, which is licensed under the Open Data Commons Open Database License (https://www.openstreetmap.org/copyright).”*

5. Please include captions for your Supporting Information files at the end of your manuscript, and update any in-text citations to match accordingly. Please see our Supporting Information guidelines for more information: [http://journals.plos.org/plosone/s/supporting-information.](https://urldefense.com/v3/__http:/journals.plos.org/plosone/s/supporting-information.__;!!NoSwA-eRAg!BtSAgaunO-NI9ok45AiYVMTB9SM7Td4gjwiYC6wu7oGyluhSgTvolsnci87HjQeD8tfyYqHuLN7tCiWsso8fT8eJdBd0AhrA$)

Thank you for pointing this out. We have separated our original supplementary-materials.docx file into S1 Table, S2 Table, S3 Table, and S1 Fig now, with their respective titles. We have also updated any reference in the main text to reflect these changes and added these captions to the end of the manuscript.

Reviewers' comments:

Reviewer's Responses to Questions

**Comments to the Author**

1. Is the manuscript technically sound, and do the data support the conclusions?

The manuscript must describe a technically sound piece of scientific research with data that supports the conclusions. Experiments must have been conducted rigorously, with appropriate controls, replication, and sample sizes. The conclusions must be drawn appropriately based on the data presented.

Reviewer #1: Yes

Reviewer #2: Partly

2. Has the statistical analysis been performed appropriately and rigorously?

Reviewer #1: Yes

Reviewer #2: Yes

3. Have the authors made all data underlying the findings in their manuscript fully available?

The [PLOS Data policy](https://urldefense.com/v3/__http:/www.plosone.org/static/policies.action*sharing__;Iw!!NoSwA-eRAg!BtSAgaunO-NI9ok45AiYVMTB9SM7Td4gjwiYC6wu7oGyluhSgTvolsnci87HjQeD8tfyYqHuLN7tCiWsso8fT8eJdA9ytb-b$) requires authors to make all data underlying the findings described in their manuscript fully available without restriction, with rare exception (please refer to the Data Availability Statement in the manuscript PDF file). The data should be provided as part of the manuscript or its supporting information, or deposited to a public repository. For example, in addition to summary statistics, the data points behind means, medians and variance measures should be available. If there are restrictions on publicly sharing data—e.g. participant privacy or use of data from a third party—those must be specified.

Reviewer #1: Yes

Reviewer #2: Yes

4. Is the manuscript presented in an intelligible fashion and written in standard English?

PLOS ONE does not copyedit accepted manuscripts, so the language in submitted articles must be clear, correct, and unambiguous. Any typographical or grammatical errors should be corrected at revision, so please note any specific errors here.

Reviewer #1: No

Reviewer #2: Yes

5. Review Comments to the Author

Please use the space provided to explain your answers to the questions above. You may also include additional comments for the author, including concerns about dual publication, research ethics, or publication ethics. (Please upload your review as an attachment if it exceeds 20,000 characters)

Reviewer #1: • In the abstract and introduction, it would be more informative if the authors could elaborate on what they mean by “burden”? in the eyes of epidemiologists, the burden could be incidence, prevalence, mortality, DALY, or a combination of them.

We included language in the abstract to clarify the definition of CC and r/mCC burden in this study:

*“We estimated CC screening rates, as well as CC burden (number of patients with CC diagnosis) and r/mCC burden (proportion of CC patients receiving systemic therapy not in conjunction with surgery or radiation),…”*

Additionally, CC and r/mCC burden descriptions are available in the in the 3^rd^ paragraph of the “Patient population and definitions” subsection in the manuscript Methods section.

• The storyline is not good through the introduction and results. The manuscript is not written in a readable style. The study has valuable data. However, this data has not been correctly directed to the objective of the study, which is to be a guide for policymakers.

Thank you for the feedback. We adjusted the introduction and results to improve the story flow and readability. We also added language in the introduction to clarify that:

- the intended audience to whom this work may be relevant include not just policymakers, but also researchers, healthcare providers, and patient advocacy groups *(“This knowledge will provide professional groups, healthcare providers, policy decision-makers and patient advocacy groups with the necessary tools to…”* in the 2^nd^ paragraph of the Introduction.)
- the envisioned use of data from this research is to provide tools for the abovementioned audience to help target areas in the US with high need for cervical cancer intervention, and also inform future research and allocation of resources to improve outcomes of patients with cervical cancer. *(“Beyond identifying areas with the highest disease burden, findings from this study may inform future research direction and targeted healthcare resources and interventions to improve outcomes for people at risk or living with CC”* as the last sentence of the Introduction.)

• Please add some previous studies reporting the possible associated factors with the disparities in CC so the reader can understand the base of your hypotheses. Or, you can provide references for line 105.

Thank you, we have included the key references that formed the basis of our hypothesis in the first paragraph of Materials and Methods.

• Results, line 176: It is not clear why “14,033 initiated a systemic therapy treatment” equals r/mCC burden”

We have corrected the phrasing from “r/mCC burden” to “r/mCC patients”. An r/mCC patient is defined in the Methods section as a CC patient that has initiated systemic therapy treatment.

• Results, the authors have the data for both screenings and sociodemographics. I wonder why they haven’t analyzed their association which would be a valuable output for this study.

Thank you for the consideration. The association between sociodemographic risk factors and cervical cancer screening have been widely studied across different populations globally including in the US and have yielded generally consistent insights into underlying drivers of disparities in screening uptake (i.e age, race/ethnicity, household income, education level, distance barriers, and disease awareness).

**United States:** Johnson NL, Head KJ, Scott SF, Zimet GD. Persistent Disparities in Cervical Cancer Screening Uptake: Knowledge and Sociodemographic Determinants of Papanicolaou and Human Papillomavirus Testing Among Women in the United States. Public Health Rep. 2020;135(4):483-91.

**Sub-saharan Africa:** Dickson KS, Boateng ENK, Acquah E, Ayebeng C, Addo IY. Screening for cervical cancer among women in five countries in sub-saharan Africa: analysis of the role played by distance to health facility and socio-demographic factors. BMC Health Serv Res. 2023;23(1):61.

**Europe:** Bozhar H, McKee M, Spadea T, Veerus P, Heinavaara S, Anttila A, et al. Socio-economic inequality of utilization of cancer testing in Europe: A cross-sectional study. Prev Med Rep. 2022;26:101733.

**China:** Zhang B, Wang S, Yang X, Chen M, Ren W, Bao Y, et al. Knowledge, willingness, uptake and barriers of cervical cancer screening services among Chinese adult females: a national cross-sectional survey based on a large e-commerce platform. BMC Womens Health. 2023;23(1):435.

However, much less is known about the potential population risk factors of r/mCC disease burden. Our study objective hence focused on the impact of demographic and socioeconomic factors, as well as availability of brachytherapy centers (as a proxy for availability of guideline-conforming care in the curative disease setting) on disease burden by US geography.

• Discussion: I was anticipating that the authors would analyze the association between HPV vaccination and CC burden across the country, but it was missing. This result would demonstrate the lack or sufficiency of this preventive measure in the shadow of the sociodemographic status.

We thank the reviewer for the suggestion on discussion of impact of HPV vaccination, which has reduced the infection rates of high-risk HPV among younger population who were eligible for vaccination. HPV vaccine was first introduced in 2006 for females aged 9-26. Over the last 18 years, the recommendation of vaccination expanded to cover females aged 9-45 and males aged 9-45. The majority of cervical cancer is associated with persistent infection of high-risk strains of HPV. While the impact of vaccination on decreased infection of HPV has been well described in literature [33], the overall incidence and mortality rates of cervical cancer have remained relatively stable over the last two decades [2]. We believe that this may be due to the combined effect of both effective screening and vaccination as the stable trend over time is more prominent among younger population (less than age 50), evident in the SEER database. We have included a short discussion on this point in the Discussion section (2^nd^ paragraph):

*“The initial introduction of HPV vaccine in 2006 and expansion of the indicated population over the decades have contributed to decrease in prevalence of the high-risk HPV infection which are more likely to lead to cervical cancer [33]. It is interesting to see that since the introduction of the vaccines, incidence and mortality rates of cervical cancer have remained relatively stable nationally [2]. This may suggest that there is a delayed effect in decreased risk of malignant disease due to the women at risk for cervical cancer today may not have been eligible for HPV vaccine at initial introduction, and that there are other risk factors, including sociodemographic status, which may confound interpretation of impact of HPV vaccination with malignant cervical cancer. Thus, in this study, we have focused on understanding association between the screening, rather than vaccination, and cervical cancer burden.”*

• Discussion: there are some previously established indices for assessing the quality of care such as healthcare access and quality (HAQ) (28528753) or quality of care index (QCI) (38273304). It is necessary for the authors to compare and justify their methodology and results (if applicable) with these indices.

Thank you for your guidance on these indices. We have not intended to create an index, as the study objective was to examine the association between disease burden and demographic and sociodemographic factors, as well as availability of brachytherapy centers by geographical units in the US. In response to an earlier suggestion by the review, we have additionally provided justification and inclusion of key references as basis for the selection of potential risk factors and analytical approach used in the present study.

Nevertheless, the HAQ index is pertinent to the discussion since the study alludes to healthcare access, and we have included the following language in second-to-last paragraph of the Discussion section:

*“Lastly, further contextualization of these insight and interpretation may be enabled by a future comparison of the CC patient population across US geographies using established indices of healthcare access, such as the Healthcare Access and Quality index [39].”*

Reviewer #2: Thank you for the opportunity to review this study. The authors leveraged claims and US Census Bureau data to assess the geographic variations in cervical cancer and poor outcomes (recurrence and metastasis) burden. They also examined the associations between cervical cancer screening rates and key variables in relation to cervical cancer burden. The study was well-designed and has interesting findings. However, I do have some questions/suggestions for improvement below.

Major issues
Introduction: The introduction is superficial and lacks information on the rationale and justification for the study. Could the author elaborate more on what previous studies have done, current gaps, and what the current study aims to add to knowledge on this specific issue?

Thank you for the feedback. We have made adjustments to the introduction to bolster the rationale and justification for our study, and more clearly articulated the specific aims and objectives of our research. We have also included more contextualization in terms of previously conducted studies on the topic and current gaps and added key reference citations. Association between sociodemographic, CC incidence, and mortality have been reported on. All previous research was done at a national level (beginning of 2^nd^ paragraph of the Introduction):

*“While healthcare disparity is well studied in prevention of CC [1-3], there is limited literature characterizing the impact of disparity on the variation in disease burden across US communities. Existing data are often focused on individual risk factors such as specific race/ethnic groups or from limited-scope registries [4-6]”*

References 8-10 all used the SEER database, while we took an approach through administrative claims.

Introduction: Please define brachytherapy at first use, for the benefit of readers who may not be familiar with the term. Also, include citations of studies that have used brachytherapy as proxy for access to guideline-concordant therapies

Thank you for the suggestion. We have included a simple definition at first mention of brachytherapy (internal radiation). Also, we included in the last paragraph of “Data” under “Materials and methods” citation to a publication “*Current standards in the Management of early and Locally Advanced Cervical Cancer: Update on the Benefit of Neoadjuvant/Adjuvant Strategies*” which directly points to the use of brachytherapy as part of the treatment guidelines for cervical cancer in the curative setting.

Methods: Could the authors expand on the Komodo Healthcare Map and what patient data are entered/included and any standardized processes guiding data inclusion? Data stated in line 109 (and presented in Table 1) is 2015-2022 different than the analyzed data 2017-2022. I recommend presenting data specific to this analysis.

Thank you. The following information about Komodo Healthcare Map were included in the 2^nd^ paragraph of “Data” under “Materials and methods”:

*“Adult patients … were identified from administrative claims data in Komodo Healthcare Map (2017-2022). The closed claims dataset includes >165 million US patients covered by Commercial, Medicaid, or Medicare Advantage plans.”*

As for the specific around type of patient data collected and processes used by Komodo to guide data inclusion, we have included a citation in the manuscript to the data vendor’s site that contains those details (including link to site: [Healthcare Map | Komodo Health](https://www.komodohealth.com/technology/healthcare-map))

Lastly, based on reviewer suggestion, we have adjusted the data presented to include only 2017-2022 data.

Methods: It appears some information is missing in lines 123 “with a diagnosis for malignant neoplasm of the cervix as identified by the ICD-123 9-CM code 180.xx or ICD-10-CM code C53.xx.”

Thank you. The term “xx” is a standard way used in ICD codes reporting to indicate that any digits can be placed there.

Methods: Please elaborate on these units of analysis and why you transformed/aggregated ZIP-5 to ZIP-3 “and race/ethnicity at the ZIP-5 level, which were then aggregated to the ZIP-3 level (6)”. Also, what proportion of enrollees were excluded due to missing ZIP-3 information?

ZIP-3 information is the most detailed location-based data we had access to, as individual-level data and count data with less than 3 people would be censored. We aggregated ZIP-5 race/ethnicity data to the ZIP-3 level so that the observation level used throughout (ZIP-3) was consistent. Artificially boosting up the number of observations we had by duplicating ZIP-3 information down to the ZIP-5 level would yield misleading statistical analysis. Less than 1% of patients had ZIP-3 information that did not map to a region (US territories or military bases), and these patients were excluded from analyses.

Results: The results section needs a major revision (Lines 197-261). Many sentences are fragmented. For example, figure labels are used in presenting results and copied verbatim in tables/ figures at the end of the manuscript.

The journal formatting guidelines requested inclusion of figure labels in-line where the figures are intended to be placed. This may have been the reason we appeared to be using figure labels to present results, though we were adhering to journal requirements. Given that these are journal requirements, we are not able to adjust this.

As for the tables/figures label at the end of the manuscript, those were not required by the journal guidelines, and we have now removed them. The duplication with the figure/table titles now longer exist.

Results: Table 1 shows data from 2015-2022 and study analyzed data from 2017 to 2022. It would be less confusing if the authors presented data specific to their analysis.

Thank you for the suggestion. We have removed any references to 2015-2016 to avoid this confusion.

Discussion: The authors should provide additional details by comparing their findings with past studies and substantiating their claims using extant data.

To the best of authors knowledge, while overall prevalence and incidence of CC and metastatic CC at national level are well described in SEER database and literature reporting analyses based on such database, this is the first study describing association of various socioeconomic and utilization of certain types of definitive treatment with disease burden in cervical cancer.

Previously, the authors have published the initial disclosure of the Geo-Analyzer tool to visualize geographical variation of cervical cancer burden. This is described in the methods section as well as now added to the Discussion section (5^th^ paragraph). This current study describes the univariate analysis of key risk factors in association with the observed disease burden with a more contemporary and comprehensive database.

Discussion: The authors introduced a new term ‘social determinants’ in the conclusion without a single mention in the preceding sections. I recommend sticking with the terms you have used throughout ‘socioeconomic’ and avoid introducing a new one in the closing paragraph.

Thank you for pointing this out. We have changed the wording from “social determinants” to “socioeconomic factors”.

Minor issues
Abstract: use ‘approximately’ instead of > unless there is a word count issue here.

Thank you, we have made this change.

Results: For the maps, can you swap the green shades such that Burden 0-15% is dark green and 15-20% is light green (similar to the dark red that signifies more serious problem)? This would enhance the visibility of the data on the maps.

Based on reviewer suggestion, we have switched the two green colors in the map and Figs 1 and S1 to reflect this change.

Results: Figures and tables are stand-alone data without a need to refer to the body of the manuscript to understand the data presented. Hence, would you please provide footnotes for each figure (and tables) offering additional important information to understand the data being presented?

Thank you. Current table and figure titles include full description of data being presented. Footnotes and legends also fully denote the outcomes data presented in the table/figures and their interpretation (where applicable), as well as full definitions for abbreviations/acronyms used.

Results and Discussion: The manuscript’s readability can be improved by organizing the results and discussion logically.

The journal formatting guidelines requested inclusion of figure labels in-line where the figures are intended to be placed. This may have been the reason we appeared to be using figure labels to present results, though we were adhering to journal requirements. Given that these are journal requirements, we are not able to adjust this.
